# Infrared Warming Reduced Winter Wheat Yields and Some Physiological Parameters, Which Were Mitigated by Irrigation and Worsened by Delayed Sowing

**DOI:** 10.1371/journal.pone.0067518

**Published:** 2013-07-09

**Authors:** Shibo Fang, Hua Su, Wei Liu, Kaiyan Tan, Sanxue Ren

**Affiliations:** 1 Institute of Eco-environment Land Agrometeorology, Chinese Academy of Meteorological Sciences, Beijing, China; 2 State Key Laboratory of Vegetation and Environmental Change, Institute of Botany, Chinese Academy of Sciences, Beijing, China; University of Illinois, United States of America

## Abstract

Winter wheat has a central role in ensuring the food security and welfare of 1.3 billion people in China. Extensive previous studies have concluded that winter wheat yields would decrease with higher temperatures, owing to warming-induced soil drying or shortening of phenophase. Temperature in China is predicted to increase by 1–5°C by 2100, which may greatly impact plant production and cause other negative effects. We performed a manipulative field experiment, creating diverse growth regimes for wheat by infrared radiation (IR) warming day and night, including IR warming only (DW), IR warming + delayed sowing dates (DS), IR warming + increased irrigation (IW), and a control (CK). The results show that IR warming increased daily average wheat canopy and soil temperatures by 2.0°C and 2.3°C, respectively. DW was associated with an advanced maturity of 10 days and yield reduction of 8.2%. IR-warming effects on the photosynthetic apparatus of wheat varied with season as well as significant differences were found in the booting stage. DS represented a worsened situation, lowering yield per plant by 16.4%, with a significant decline in aboveground biomass and functional leaf area. Wheat under DS showed double-peak patterns of diurnal gas exchange during booting stages and, consequently, lower photosynthetic capacity with high transpiration for cooling. Significantly lower actual water use efficiency and intrinsic water use efficiency from jointing to anthesis stages were also found under DS. However, IW had no significant difference from CK, irrespective of yield and photosynthesis. Therefore, we concluded that delayed sowing date may not be a good choice for winter wheat, whereas a thoroughly-watered wheat agroecosystem should be promoted in the context of global warming.

## Introduction

Global mean surface air temperature increased by about 0.5°C during the 20^th^ century, and is projected to increase further by 1.8°C to 4.0°C by the end of this century [1]. Temperature in China has increased by 1.2°C since 1960, and is estimated to increase further by 1–5°C by 2100 [2]. Warming will greatly impact plant production [3], [4], and additional negative effects have been found in temperate crop cultivation areas. Ottman et al. (2012) reported that grain yield of wheat declined 6.9% per 1°C increase in seasonal temperature above 16.3°C at Maricopa, AZ [5]. Mitchell *et al*. (1995) found that warming by 4.0°C reduced winter wheat phytomass and yield by 20–30% in the UK [6]. Asseng *et al*. (2010) observed variations in average growing-season temperatures of ±2°C in the main wheat growing regions of Australia, and found reductions in grain production up to 50% [7]. Fang *et al*. (2010) found that nocturnal warming of 2.5°C caused 27% declines of winter wheat yield in northern China [8]. Long-term observations [9], [10], manipulative experiments [11] and model simulations [12] have also demonstrated negative impacts of rising temperatures on biomass production and yield of other crop species. However, underlying mechanisms of crop response to climatic warming and consequent influences on crop biomass production and yield remain elusive.

Winter wheat is the foremost cereal-grain food in the world and an essential source of carbohydrates [13], especially in China. It has a central role in ensuring the food security and welfare of 1.3 billion people. Several studies have found that reduction in winter wheat yields with higher temperatures may be due to warming-induced soil drying [12], [13] or shortening of phenophase [8]. Warming-induced change in growth environments would certainly affect crop-water relationships [14], [15] and/or photosynthesis [13], [16], [17], which are the principal determinants of total crop biomass [18]. Today, because of little effort toward increasing the proportion of biomass allocated to grains, increasing total crop biomass may be more important for achieving greater yields [18]. Therefore, it is essential to investigate the effects of warming on photosynthetic capacities and wheat biomass, and to find countermeasures to prevent their reduction.

The use of infrared radiation (IR) heaters to conduct ecosystem warming experiments *in situ* has been recently developed [13], [19]–[22]. Hence, we conducted a manipulative field experiment, creating diverse growth regimes for experimental wheat by IR warming only, delayed sowing dates + IR warming, or increased irrigation + IR warming. Our primary objective was to compare variations in photosynthesis and biomass of wheat during the same growth stage, i.e., jointing, booting and anthesis. We hypothesized that warming would result in significant down-regulation of photosynthetic capacity, which would induce significant reduction of wheat biomass. However, delaying sowing time and/or increasing irrigation might alleviate the negative effects.

## Materials and Methods

### Study site

The research site was at the Gucheng Agrometeorological Experimental Center of the China Meteorological Administration (39°08′N, 115°40′E, 15.2 m a.s.l.) in Dingxing County, Hebei Province. The site has a typical cinnamon soil. Soil bulk density and pH are 1.35 g cm^−3^ and 8.1, respectively. Soil organic C, total N, available P and available K content are 13.67 g kg^−1^, 0.87 g kg^−1^, 25.76 mg kg^−1^ and 118.55 mg kg^−1^, respectively. Meteorological data were obtained from the Baoding weather station. Mean annual precipitation is 551.5 mm, with 87% between May and September. Mean annual temperature is 11.7°C, with minimum and maximum temperatures −8.8°C in January and 22.2°C in July, respectively.

### Crop culture

Winter wheat (*Triticum aestivum* cv. super-626) was selected as the experimental material. Three treatments were randomly arranged, i.e., IR warming both day and night (DW), delayed sowing date plus warming (DS), increased water addition together with warming (IW), and a control (CK – without warming; normal sowing and watering), four replicates for each treatment. Seeds under CK, DW and IW were sown on 10 October 2008, and under DS on 25 October 2008. Wheat was planted in 2 m×4 m plots separated by 2 m alleyways. Soil fertility was managed to avoid nutrient limitations. A preplant fertilizer mix was applied, consisting of urea (N 40%; 200 g/plot) and compound fertilizer (N 22%, P 10%, K 16%; 400 g/plot). Nitrogen fertilizer was subsequently applied in irrigation water as urea (N 40%; 300 g/plot). Six irrigations were executed as needed; CK, DS and DW irrigation amounts (controlled by electronic control water meter) were the equivalent of 100 mm (0.8 m^3^ per plot) precipitation, whereas IW received 20 mm (0.16 m^3^ per plot) more water of each irrigation. No supplemental irrigations were applied for the heated plots, even though the evaporation should be increased as described by Kimball (2005, 2011) [20], [23] and Wall et al. (2011) [13]. Any natural precipitation was blocked by a large-scale moveable rain shelter. All other agronomic activities were in accord with local recommended practices.

Guidelines for phenological observations and yield component measurements were according to agro meteorological observation standards [24]. Onset of anthesis was recorded when 50% of spikes had at least one visible anther [25].

### Infrared radiator warming apparatus

**Figure 1:**
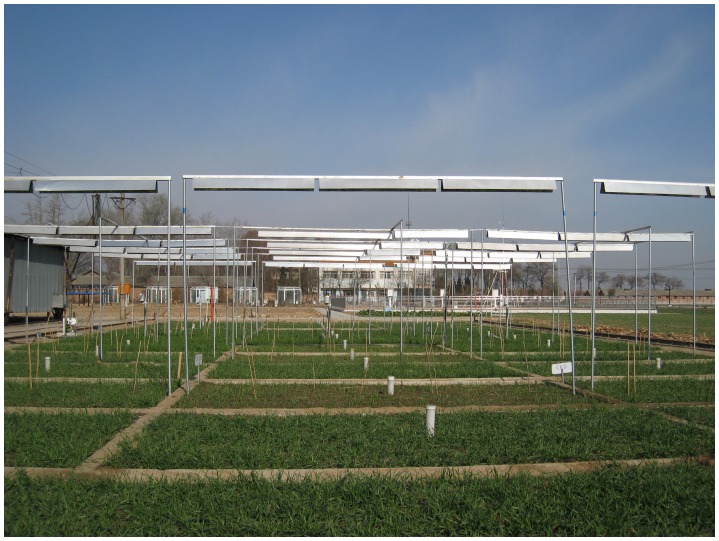
View of heated plots on 10 March 2010 at Hebei province, China.

Three infrared radiator heaters (Beijing Sanyuan Huahui Lighting Ltd., Beijing Lighting Research Institute; 500 W, 220 V, 1300 mm long ×12 mm wide) were fixed in a semicircle stainless-steel mirror-reflector for each IR-warming plot, according to Wan *et al*. [22]. The heaters were suspended at a height of 2.3 m above the ground (Fig. 1) and were set at a radiation output of ∼1500 W. Simultaneously, specular reflection lampshades were added to avoid energy loss. Three “dummy” heaters of the same shape and size as the infrared heater was used for each CK plot to simulate the shading effect. The DW, DS and IW plots were heated by infrared radiator heaters during either daytime or nighttime, from seed sowing to grain harvest. Target warming levels were 2–3°C higher canopy air temperature.

### Temperature measurements

Canopy air temperatures (Tc) measurements were taken with factory calibrated thermometer sensor (Model HC2S3-L, Campbell Scientific Inc., Logan, Utah, USA) at canopy height (which was gradually increased according to plant height). The sensors were mounted inside of a naturally ventilated radiation shield (type 439101, Feingerätebau K. Fischer GmbH, Germany), protected from direct sunlight. However, because wind speeds are reduced at canopy height, it is possible that ventilation was inadequate, and therefore, the heaters may have warmed the sensors more than they warmed the air.

Soil temperature (Ts) was recorded automatically on-site using factory calibrated thermometer sensors (HMP107, Campbell Scientific Inc., Logan, Utah, USA). The sensor was buried at 20 cm below the soil surface in the middle of the plot before sowing.

Temperature measurements were taken every 1 min and the averages of the sixty measurements within 1 h were stored as the hourly means. Daily maximum and minimum temperatures were calculated by hours.

### Gas exchange measurement

Five plants per treatment were sampled when the wheat plants reached the jointing, booting and anthesis stages. Gas exchange was measured on the most recently fully-expanded (emerged ligule) sunlit leaves on the main plant stem, using a portable gas exchange system (Li-6400; LiCOR Inc., Lincoln, NE, USA). Each measurement was repeated six times in six days. The monitor was run at 06∶00, 09∶00, 11∶00, 13∶00, 15∶00 and 18∶00 (local time) with temporal photosynthetic photon flux density (PAR). Air flow rate was set at 500 µmol min^−1^, and CO_2_ concentration in the leaf cuvette was maintained at 400 µmol mol^−1^, which was approximately the same as CO_2_ concentration near the plant canopy. Ambient temperature, atmospheric vapour pressure deficit (VPD), intercellular CO_2_ concentration (*C_i_*), transpiration rate (*E*) and stomatal conductance (*g_s_*) were simultaneously recorded with the assimilation rate (*A*) from 06∶00 to 18∶00. Additionally, actual and intrinsic water use efficiencies (*WUE*) were calculated as *A*/*E* and *A*/*g_s_*, respectively [13].

### Dry matter accumulation and partitioning

A total of 1 m^2^ plants per plot were harvested for yield, and 10 plants per plot for yield components. The grains from each ear of wheat were threshed by hand and the number of grains per ear counted. The yield per ear, yield per plant and seed size (1000 seed weight) were determined after drying seeds in the sun. Three plants per plot were portioned into stem, leaf, ear and grain, and dried to constant weight in an oven at 72°C for at least 48 h.

### Statistical analysis

The experiment was randomized complete block design with a single factor (treatment). The seasonal variations of T_c_, T_c,max_, T_c,min_, T_c,avr_, T_cd,avr_, T_cn,avr_, T_s_, T_s,max_, T_s,min_, T_s,avr_, T_sd,avr_ and T_sn,avr_ in heated and unheated plots were obtained by using linear regression analysis, respectively. One-way analysis of variances (ANOVAs) were used to determine significant differences (*P*<0.05). The seasonal *A*, *g_s_*, *E*, *C_i_* and VPD of each treatment at each stage was calculated from averaging diurnal gas exchanges except of the values at 6∶00 hour and 18∶00 h. ANOVAs were used for seasonal changes to examine the effects of different treatments. Two-way ANOVAs were used for diurnal changes to examine the effects of different treats and times. Differences between treatments were considered significant at *P*<0.05 following least significant difference (LSD) test. All statistical analyses were conducted with the SPSS software version 18.0 (SPSS, Chicago, IL, USA).

## Results

### Infrared warming

**Figure 2:**
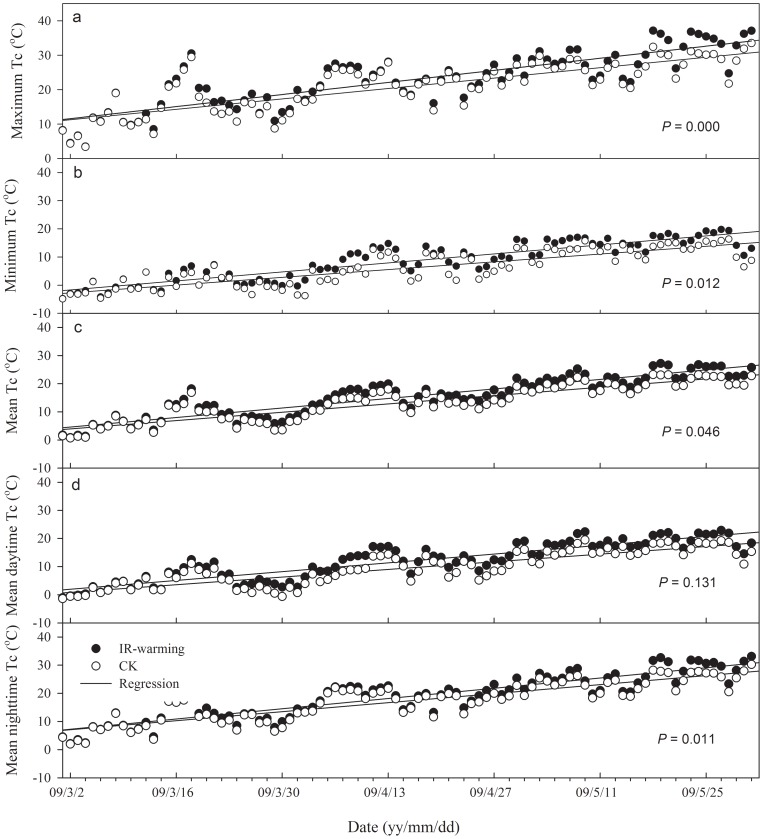
**Comparison of winter wheat canopy temperature (** ***T_c_*** **) between IR-warming and control (CK) plots.** *T_c,max_*, the maximum daily canopy temperature; *T_c,min_*, the minimum daily canopy temperature; *T_c,avr_*, the average daily canopy temperature; *T_cd,avr_*, the average canopy temperature during daytime; *T_cn,avr_*, the average canopy temperature during nighttime; *P* value are used to show significance. *P*<0.05 indicated significant difference between regressions.

**Figure 3:**
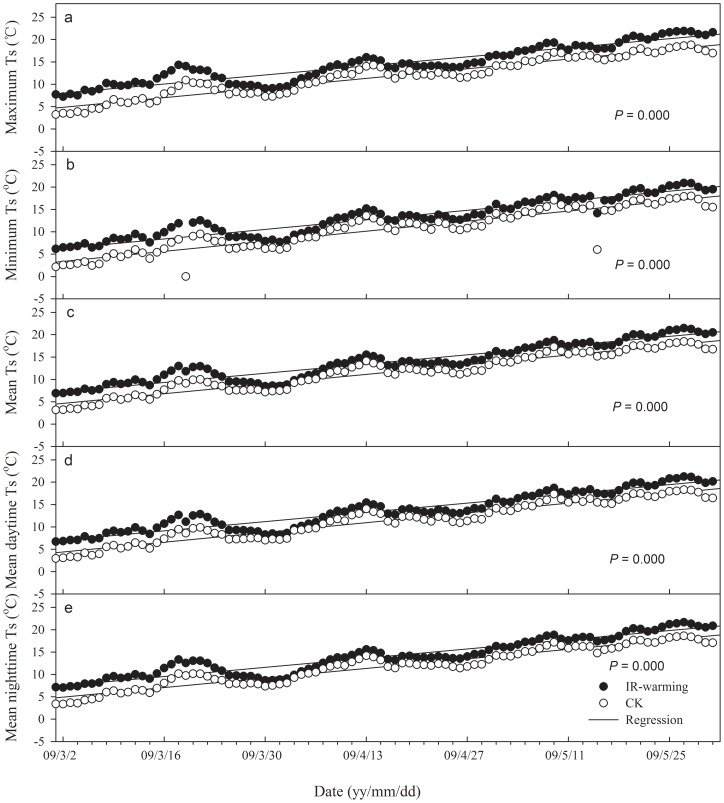
Comparison of soil temperature (*T_s_*) between IR-warming and control (CK) plots. *T_s,max_*, the maximum daily soil temperature; *T_s,min_*, the minimum daily soil temperature; *T_s,avr_*, the average daily soil temperature; *T_sd,avr_*, the average soil temperature during daytime; *T_sn,avr_*, the average soil temperature during nighttime; *P* value are used to show significance. *P*<0.05 indicated significant difference between regressions.

Over the study period, overall average significant increases were found in wheat T_c_ (Fig. 2) and T_s_ (Fig. 3) when IR warming was added, with values of 2.0°C (Fig. 2C) and 2.3°C (Fig. 3C), respectively. The maximum and minimum T_c_ of heated plots were significantly higher than those of CK, as were the maximum and minimum T_s_. Mean daytime T_c_ under warming was 12.6°C (with a range from −1.0°C to 22.3°C), and had no significant difference with CK (Fig. 2D). Mean daytime T_s_ under warming was 13.6°C (with a range from 6.7°C to 20.8°C; Fig. 1D), 2.3°C higher than CK (Fig. 3D). The mean nighttime T_c_ under warming was 19.8°C (with a range from 4.5°C to 29.2°C), 1.6°C higher than CK (Fig. 2E). The mean nighttime T_s_ under warming was 14.0°C (with a range from −6.7°C to 20.8°C; Fig. 2D), 2.3°C higher than CK (Fig. 3E).

**Figure 4:**
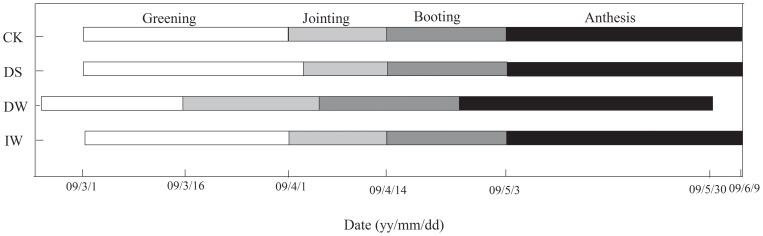
Growth stages of four treatments, i.e. control (CK), delay sowing date as well as warming (DS), warming both day and night (DW), increase water addition as well as warming (IW).

Winter wheat under DW tended to reach maturity much earlier than other plots, by about 10 days (Fig. 4). However, both DS and IW could alleviate any differences, and both reached maturity simultaneously with CK.

### Yield and yield attributes

Compared with CK, DW drastically reduced yield (9.0%) and seed size (21.2%) of winter wheat. DW also increased ear biomass per plant and seeds per ear, but had no effects on yield per year or aboveground biomass per plant (Table 1). Under DS, yield was reduced by 16.2%; ear biomass per plant, yield per ear and aboveground biomass per plant also decreased (Table 1). However, under IW, there was no noteworthy difference for all yields and yield attributes, except of ear biomass (Table 1).

**Table 1:** Yield and yield attributes for a winter wheat, *Triticum aestivum* cv. super-626, to different treatments, i.e. control (CK), delay sowing date as well as warming (DS), warming both day and night (DW), increase water addition as well as warming (IW). Mean (± SEM). *n* = 5.

Treatments	Ear biomass /Plant (g)	Seeds/Ear (No.)	Seed Size (g/1000seeds)	Yield/Ear (g)	Yield (g/m^2^)	Aboveground biomass/plant (g)
CK	2.16±0.03^b^	31.60±1.15^b^	41.78±0.47^a^	1.10±0.02^a^	712.23±36.09 ^a^	3.13±0.01^a^
DS	2.08±0.02^c^	33.23±0.64^b^	40.79±1.29^a^	0.97±0.03^b^	596.59±27.28^bd^	2.86±0.03^b^
DW	2.36±0.01^a^	37.90±0.87^a^	32.91±1.16^b^	1.07±0.03^a^	648.07±45.16^bc^	3.21±0.06^a^
IW	2.24±0.08^a^	33.13±0.48^b^	41.55±0.79^a^	1.13±0.04^a^	694.7±39.07 ^a^	3.23±0.05^a^

### Biomass and functional leaves

**Figure 5:**
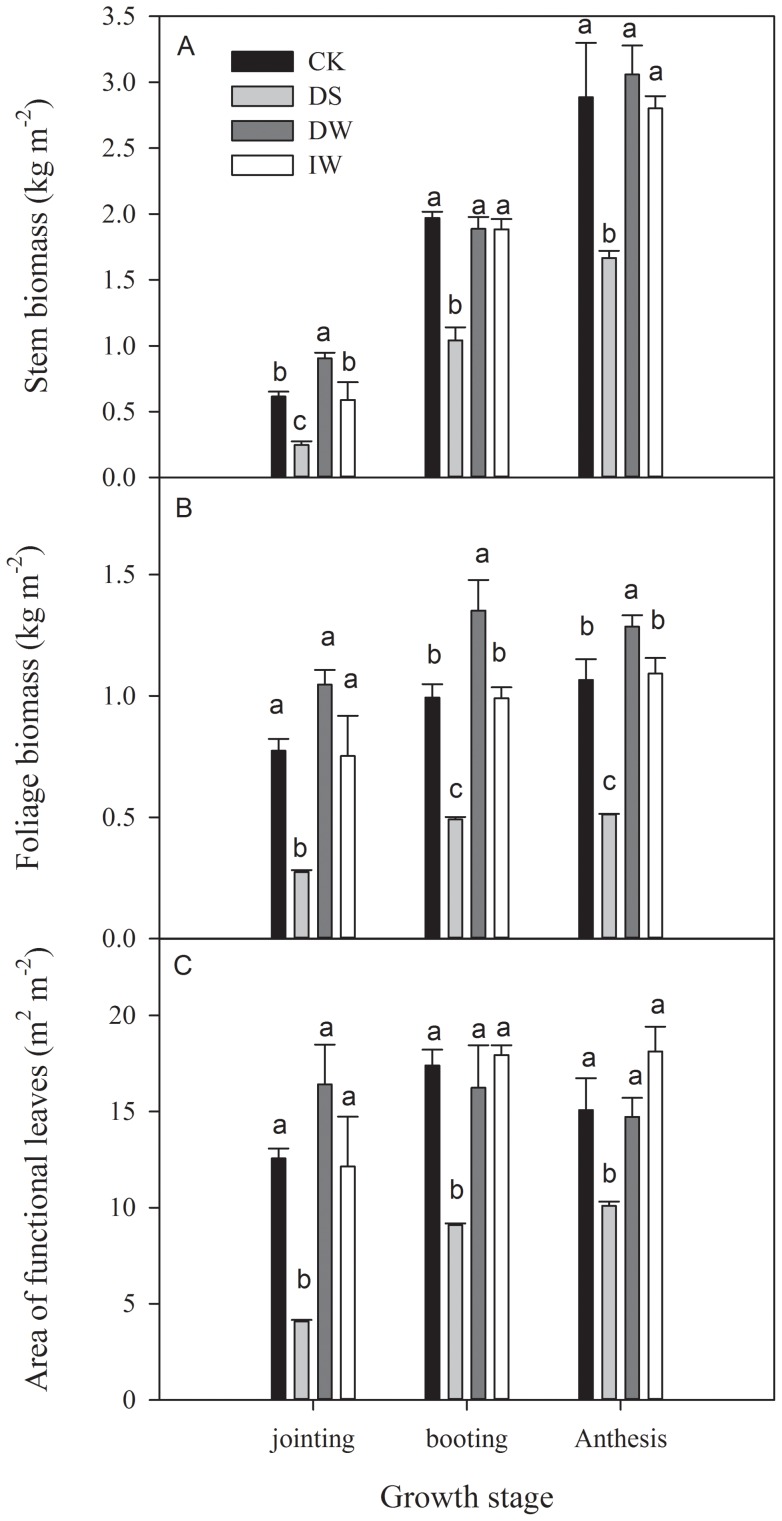
Season changes in (A) stem biomass, (B) foliage biomass and (C) areas of functional leaves of winter wheat under four treatments (CK, DS, DW and IW). Data were calculated based on 1 m^2^ soil area. Different lower case letters indicated significant difference (*P*<0.05).

Stem biomass of winter wheat gradually increased through the experimental periods, with and without IR heating, with DS always lowest; it was 42.3% lower than CK during the anthesis stage (1.67 kg m^−2^) (Fig. 5A). There were two types of growth trend in foliage biomass among the four treatments (Fig. 5B). DS, IW, and CK maintained an increasing trend from jointing to anthesis stage (Fig. 5B). DW peaked (1.35 kg m^−2^) at booting stage and then decreased. There were no significant differences of functional leaf area during experimental periods for DW, IW and CK, but DS was lower (Fig. 5C). At the anthesis stage, DS was lower by 33.1%, 31.5% and 44.3% relative to CK, DW and IW, respectively (Fig. 5C).

### Diurnal patterns of gas exchange

**Figure 6:**
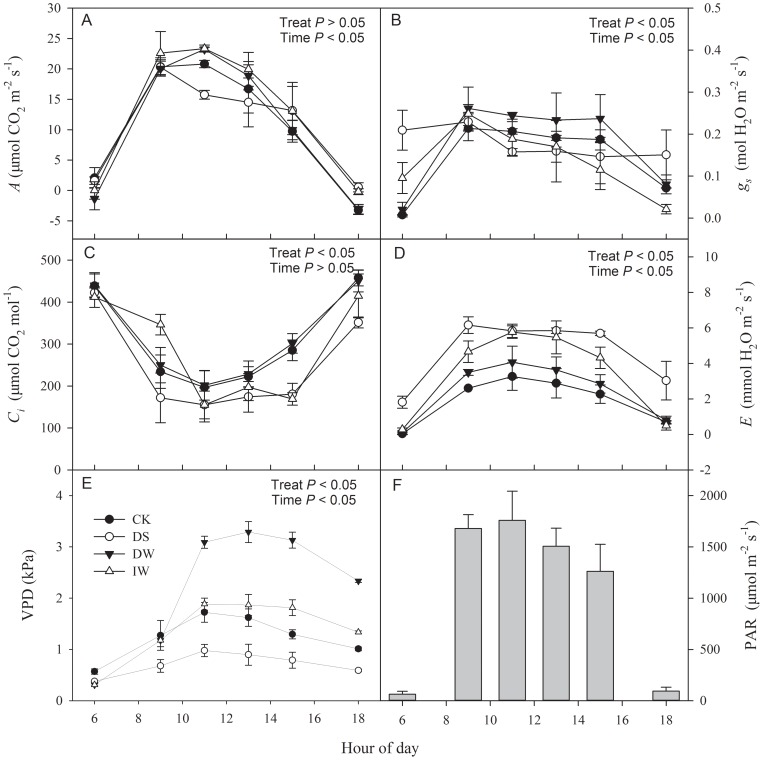
Diurnal changes in (A) assimilation rate (*A*), (B) stomatal conductance (*g_s_*), (C) intercellular CO_2_ concentration (*C_i_*), (D) transpiration rate (*E*) and (E) atmospheric vapour pressure deficit (VPD) (mean ± SE) in winter wheat under four treatments (CK, DS, DW and IW), as well as (F) photosynthetic photon flux density (PAR) in jointing stage. *P* value represent the significance of the effects of each treatment.

**Figure 7:**
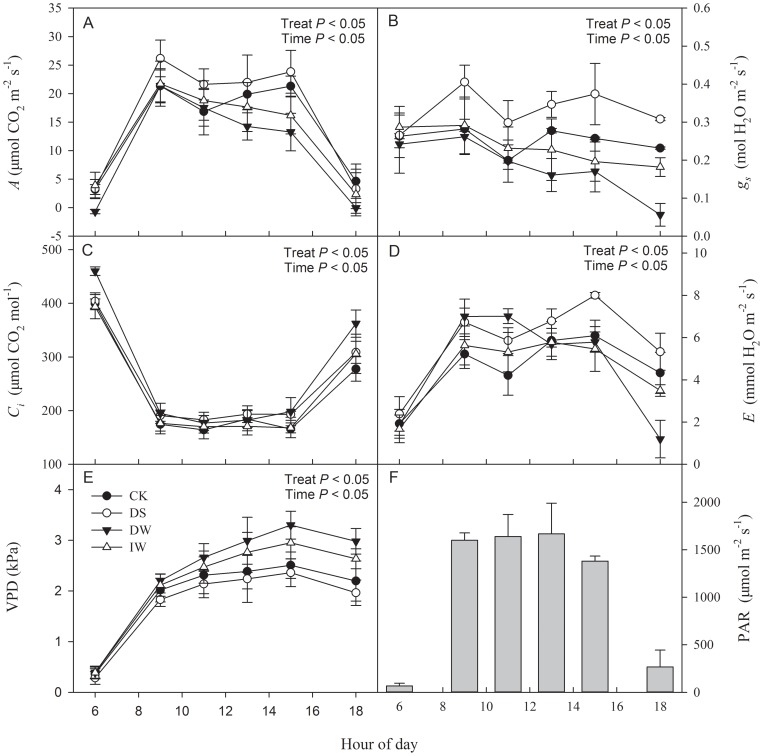
Diurnal changes in (A) assimilation rate (*A*), (B) stomatal conductance (*g_s_*), (C) intercellular CO_2_ concentration (*C_i_*), (D) transpiration rate (*E*) and (E) atmospheric vapour pressure deficit (VPD) (mean ± SE) in winter wheat under four treatments (CK, DS, DW and IW), as well as (F) photosynthetic photon flux density (PAR) (E) in booting stage. *P* value represent the significance of the effects of each treatment.

**Figure 8:**
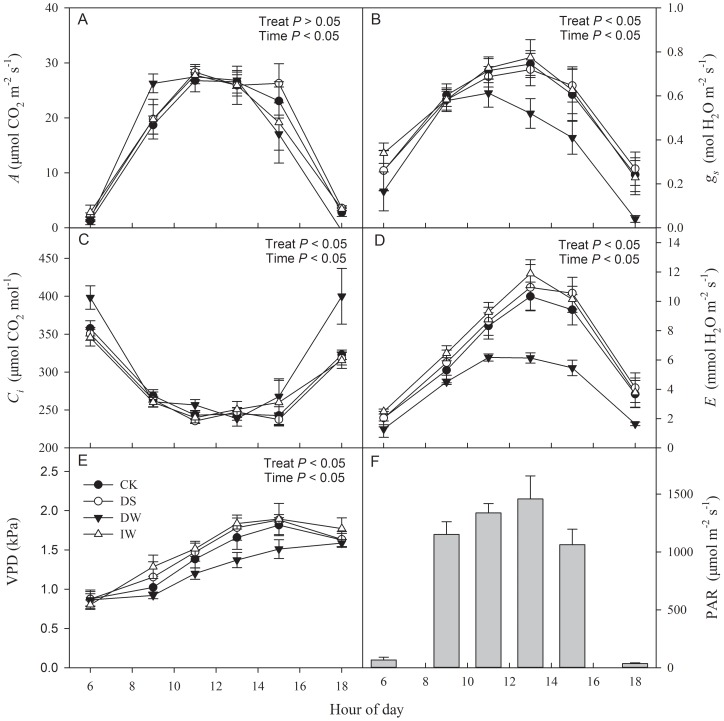
Diurnal changes in (A) assimilation rate (*A*), (B) stomatal conductance (*g_s_*), (C) intercellular CO_2_ concentration (*C_i_*), (D) transpiration rate (*E*) and (E) atmospheric vapour pressure deficit (VPD) (mean ± SE) in winter wheat under four treatments (CK, DS, DW and IW), as well as (F) photosynthetic photon flux density (PAR) (E) in anthesis stage. *P* value represent the significance of the effects of each treatment.

In the study area, diurnal patterns of PAR were similar during the growth season of winter wheat (Figs. 6F, 7F and 8F). After sunrise at approximately 06∶00, PAR increased rapidly, peaked between 09∶00 and 15∶00 (with a range from 1200–1800 μmol m^−2^ s^−1^), and decreased thereafter. With IR heating or no heating, winter wheat showed similar diurnal patterns of photosynthesis during jointing, booting and anthesis stages (Figs. 6, 7 and 8). However, there were some differences between warming treatments and CK.

At the jointing stage, the maximum value of *A* for either DW (23.2 μmol m^−2^ s^−1^) or IW (23.4 μmol m^−2^ s^−1^) was higher than CK (20.8 μmol m^−2^ s^−1^), all of which appeared at 11∶00. However, the diurnal patterns of *A* under CK, DS, DW and IW had no significant differences (*P*>0.05), performing as unimodal curves. There were significant differences in diurnal *g_s_* changes of winter wheat for CK, DS, DW and IW, and the peak value of *g_s_* (around 0.2 mol H_2_O m^−2^ s^−1^) for IW, DW and DS was higher than CK (Fig. 6B). The *g_s_* of IW dramatically decreased after 09∶00, while CK, DW and DS remained relatively stable. The *E* of DS and IW were significantly higher than CK (Fig. 6D). The *C_i_* showed opposite trends with *A* or *g_s_* or *E*. The *C_i_* of DS and IW were significantly lower than CK, while that for DW was almost the same (Fig. 6C). The VPD of DW were significantly higher than CK, especially after 9∶00 hour, while that of DS were significant lower than CK (Fig. 6E).

At booting stage, diurnal changes of *A*, *g_s_* and *E* showed double peaks (9∶00 and 15∶00) under both CK and DS, with a single peak (9∶00) under both DW and IW (Fig. 7). Moreover, the values of *A*, *g_s_* and *E* under DS were the highest of the four treatments, whereas those of DW were the lowest (Fig. 7A, B and D) (*P*<0.05). There were trough patterns of *C_i_* in the four treatments (Fig. 7C), and that in DW was significantly higher. Diurnal changes of VPD showed the same trend under the four treatments but with significant differences among the values following the sequence of DW > IW > CK > DS (Fig. 7E).

At the anthesis stage, diurnal changes of *A*, *g_s_* and *E* showed single-peak curves and the peak value appeared at 11∶00. There were no significant differences of *A* among the four treatments (Fig. 8A), and the maximum value was about 25.0 μmol m^−2^ s^−1^. The *g_s_* of DW was significantly lower than CK, while those in DS and IW were basically in accord with CK (Fig. 8B), as was the case for *E* (Fig. 8D). In contrast, the *C_i_* of DW was significantly higher than CK, while those in DS and IW changed correspondingly with CK (Fig. 8C). The VPD of DW was significantly lower than CK, while those in DS and IW were significantly higher than CK (Fig. 8E).

### Seasonal patterns of gas exchange

**Figure 9:**
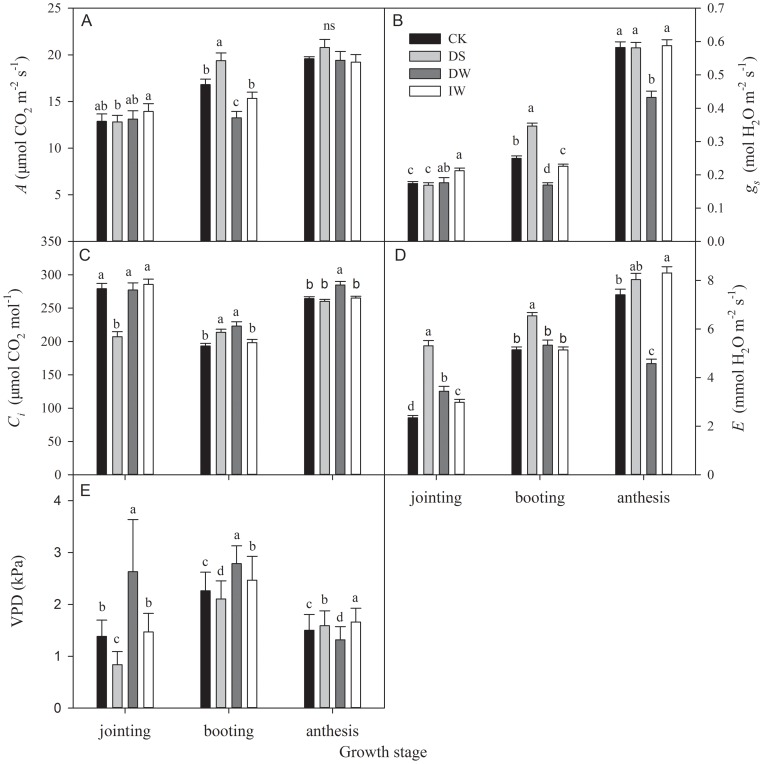
Seasonal changes in (A) assimilation rate (*A*), (B) stomatal conductance (*g_s_*), (C) intercellular CO_2_ concentration (*C_i_*) (D) transpiration rate (*E*) and (E) atmospheric vapour pressure deficit (VPD) (mean ± SE) in winter wheat under four treatments (CK, DS, DW and IW), during growth stages. Significant differences (*P*<0.05) are indicated with different lower case letters.

Greater or lower values of gas exchange of wheat in IR-warming plots were observed from jointing stage to harvest. Seasonal changes of *A*, *g_s_* and *E* in CK increased substantially. Compared with CK during booting stage, DW had significant effects on gas exchange (Fig. 9). Under DW, there was a 21.1% lower *A* (16.81 μmol m^−2^ s^−1^) (Fig. 9A), *g_s_* was significantly lower by 32.1% (Fig. 9B), *C_i_* was significantly higher by 15.5% (Fig. 9C) and VPD was significantly higher by 23.2% (Fig. 9E); *E* had no significant differences (Fig. 9D) during booting stage. However, it was noteworthy that *E* for DW was significantly lower by 38.2% compared with CK during anthesis stage. Distinguishable variables of gas exchange between DS and CK were also found during booting stages; significantly higher *A*, *g_s_*, *C_i_* and *E* were observed, by 15.3%, 38.9%, 10.6% and 27.2%, respectively. Nearly identical variables were obtained between IW and CK.

### Water use efficiency

**Figure 10:**
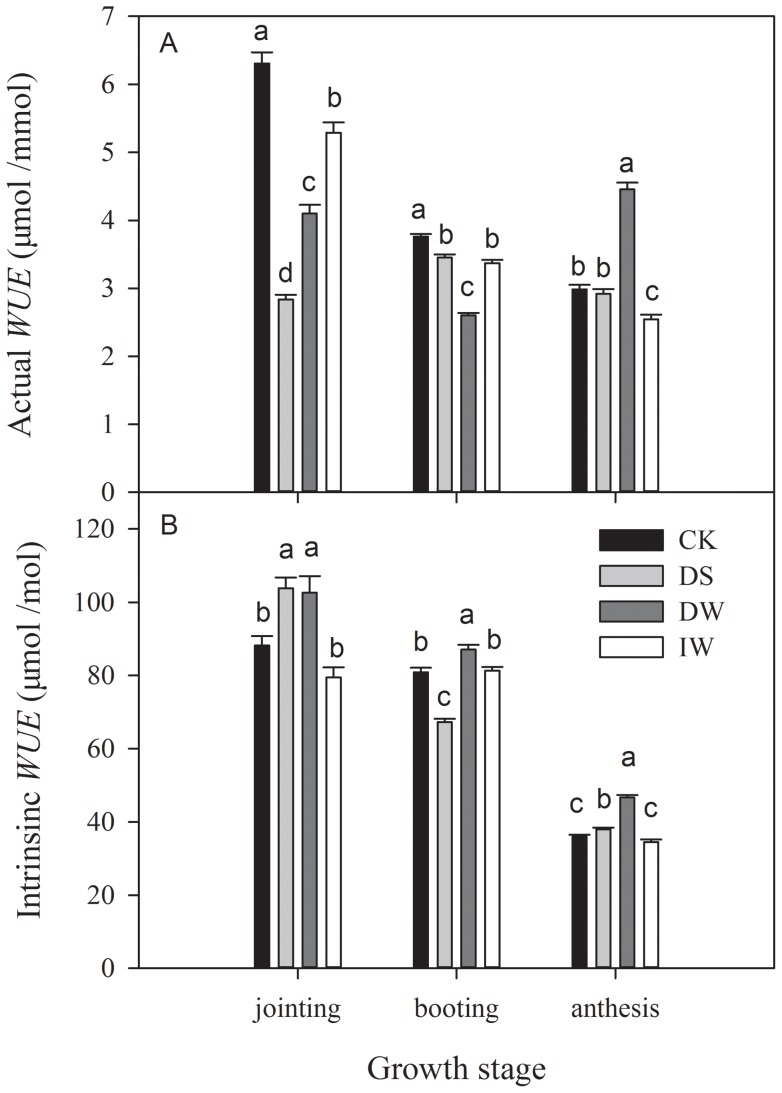
Seasonal changes in (A) actual water use efficiency (*WUE*) and (B) intrinsic *WUE* in winter wheat under four treatments (CK, DS, DW and IW), during growth stages. Significant differences (*P*<0.05) are indicated with different lower case letters.

Different trends were observed for actual *WUE* among the four treatments (Fig. 10A). CK showed a decreasing trend (from 6.31 to 2.99 μmol mmol^−1^), as did IW (from 5.29 to 2.54 μmol mmol^−1^). DS had a peak (3.45 μmol mmol^−1^) while DW had a trough (2.60 μmol mmol^−1^) during booting stage. There were significant differences between heated treatments and CK during booting and anthesis stages (Fig. 10B). Compared with CK, there was 7.6% higher intrinsic *WUE* of DW and 16.8% lower of DS during booting stage, and significantly higher Intrinsic *WUE* values of DW (46.62 vs 35.96 μmol mol^−1^) and DS (38.03 vs 35.96 μmol mol^−1^) during anthesis stage. There were no significant differences between CK and IW throughout the experimental periods.

## Discussion

### IR warming in the field

IR warming increased average wheat T_c_ by 2.0°C, however, there was no significant increase during daytime and a 1.6°C increase at night, relative to CK (Fig. 2). Although the heaters were fully operational, average temperature hardly rose above that of CK during daytime, except of the height of the heaters related to the canopy, perhaps mainly because the heaters could not supply enough heat during windy periods, as reported by Wall *et al.* (2011)[13] and Ottman *et al*. (2012) [5]. A more powerful heating system, such as described in Kimball *et al.* (2008) [19], is needed to attain significant warming effects of wheat Tc in the field, during night and day. However, maximum Tc of IR-warmed plots was significantly higher than CK in this study (Fig. 2).

Continuous warming significantly increased mean soil temperature at 20 cm depth. Average T_s_ of heated plots was 2.3°C higher than CK; both daytime average T_s_ and nighttime average T_s_ were also higher by 2.3°C.

### IR warming-induced maturity advance and yield reduction of wheat

In our experiments, high temperatures were associated with advanced maturity and reduced yield (Table 1 and Fig. 5). IR warming caused about a 10-day maturity advance and 8.2% decrease in the yield of wheat (Table 1). Identical results have been reported [26]–[28]. In northern Europe, soil warming hastened crop development during early stages and shortened the crop growing season by 12 days [29]. For the southern UK, Butterfield and Morison (1992) predicted that mean warming of 2°C or 4°C would reduce the duration of a winter crop by 20 or 35 days, and reproductive phase by 8 or 20 days, respectively [30]. IR warming increases plant organ temperature and accelerates plant growth and development [13], because a T_c_ increase can have a considerable cumulative effect [31]. Zhang and Huang (2012) suggested that an increase in maximum T_c_ appeared to be the most important reason for yield reduction of wheat in northeast China [12]. Moreover, higher T_s_ may cause a drier environment for plants [13], [15], [32], [33] which hasten the rate of wheat senescence [34] and significantly limits wheat yields [35] because drought decreased photosynthesis and viable leaf area [36].

Increased temperatures cause premature plant senescence and shorten the period of photosynthetic capacities [26]. Warming effects on photosynthetic rates remain controversial [37]. Some studies have reported no changes in these rates [32], [38]–[40], but others have found decreases [41] or increases [42], [43]. During jointing and anthesis stages, no significant difference of diurnal patterns of *A* were found between DW and CK (Fig. 6A); even the average value of maximum *A* had no significant difference (*P*>0.05) (Fig. 9A). However, there were significant differences between DW and CK during booting stage, irrespective of diurnal trends or average maximum values (Figs. 7A and 9A). These results indicate that IR-warming effects on the photosynthetic apparatus of wheat varied with season. This agrees with the findings of Niu et al. (2011), who demonstrated that thermoregulation of this apparatus only occurred during the mid-growing season, and not in its early or late portions [43]. However, Zhou et al. (2007) indicated that the effect of IR warming on leaf photosynthesis rates varied with species [42]. Both up- and down-regulation of the optimal temperature for photosynthesis suggested that photosynthetic acclimation was species-specific. Our results were inconclusive regarding detection of a thermal acclimation response of optimal temperature for wheat to warming trends.

IR warming readily causes heat stress accompanied by drought stress, which presumably accelerates aging [44]. Higher water depletion affected *g_s_* and *E* (Figs. 6 to 9), which likely diminished transpiration cooling of the leaf, especially during the anthesis stage. IR-warming affected plant-water relations across a variety of species [33], and our results for the wheat crop are in agreement. A higher Intrinsic *WUE* of DW than CK was observed in this study (Fig. 10B).

### Serious reduction of yield from delay in sowing date and warming

For winter wheat in northern China, the recommended sowing date is at the beginning of October [45]. Sowing dates later than the recommended dates may result in reduced growth and grain yield [46]. The yield for DS in this study were found to be lower than CK by 16.4%. Ear biomass per plant, yield per ear and aboveground biomass per plant were also significantly decreased (Table 1 and Fig. 5). Additionally, late sowing significantly decreased stem biomass, foliage biomass, and areas of functional leaves, which consistent with Royo *et al*. (2000) [47]. White et al. (2011) suggested that the growth and yield reduction due largely to decreased tiller number, shorter overall growth cycle, and high temperatures during later stages [24]. Royo et al. (2000) reported that delayed sowing date significantly reduced grain filling duration and final grain weight [47].

In wheat, phenology is especially sensitive to temperature, reflecting both a direct response of intrinsic development rate and a response mediated by vernalization processes [31], [48]. Higher temperature cause a faster plant development (shorter growing season), which decreased the photosynthesis period and hampered full grain filling [49]. In this study, DS and CK entered booting stage at the same time in this study although DS sowing seed later 15 days than CK (Fig. 4). There have no significant differences of photosynthetic capacities between DS and CK during both jointing and anthesis stages (Fig. 9), even with significant higher *A* and *E* of DS than CK during booting stage.

### Compensation for the negative effects of warming by increasing irrigation

Yield and yield attributes of IW had no significant difference with CK (Table 1 and Fig. 5) while DW had a significant reduction. Significant higher vapour pressure deficit (VPD) were found in DW during both jointing and booting stage (Fig. 6E and Fig. 7E), which implied that IR-warming tends to increase water loss from plants and the soil surface [50]. As reported, warming reduced annual soil water content by 13.1% in a semi-arid grassland [51]. Although water loss in general is lower in moist than in dry air, special caution was also suggested when using IR heaters in humid conditions [50]. In this study, the difference of VPD between IW and CK were smaller than that between DW and CK. Differences of gas exchange and *WUE* between IW and CK were also minor (Figs. 6 to 10). So, the excess water loss and negative effects under IR-warming in the field experiment could be compensate by increasing irrigation, at least under the limited conditions of our experiment, which have also been proved by De Boeck *et al*. (2012) [50] and Wall *et al*. (2011) [13].

However, further study should be taken to verify two quantitative amounts: the supplemental amounts to offset the additional evapotranspiration and the irrigation amounts to compensate for negative effects of warming. Kimball et al. (2005) had reported that the supplemental amounts are 6.3% times the evapotranspiration from the reference plot per Celsius of warming [20]. De Boeck et al. (2012) suggested a 12–15% increase in transpiration under infrared heaters for a 1°C warming in temperate climate [50]. Therefore, it is necessary to irrigate additional water for the heated plots to account for the increased evaporation and make the infrared heater treatments more equivalent to air heating at constant relative humidity as described by Kimball (2005, 2011) [20], [23] and Wall *et al*. (2011) [13].

## Conclusion

Our results demonstrate the following. 1) Climate warming significantly reduced wheat growth and yield in northern China by shortening growing seasons, and perturbed leaf photosynthesis at critical times, such as the booting stage. 2) Delaying sowing dates, which will change phenology and cause a faster plant development, may not be a good choice for winter wheat in the context of global warming. 3) A thoroughly-watered wheat agroecosystem is recommended under IR warming conditions representative of some future point in a global warming trajectory.
